# BioGD: Bio-inspired robust gradient descent

**DOI:** 10.1371/journal.pone.0219004

**Published:** 2019-07-05

**Authors:** Ilona Kulikovskikh, Sergej Prokhorov, Tomislav Lipić, Tarzan Legović, Tomislav Šmuc

**Affiliations:** 1 Department of Information Systems and Technologies, Samara National Research University, Samara, Russia; 2 Faculty of Electrical Engineering and Computing, University of Zagreb, Zagreb, Croatia; 3 Division of Electronics, Ruđer Bošković Institute, Zagreb, Croatia; 4 Division of Marine and Environmental Research, Ruđer Bošković Institute, Zagreb, Croatia; University of Rijeka, CROATIA

## Abstract

Recent research in machine learning pointed to the core problem of state-of-the-art models which impedes their widespread adoption in different domains. The models’ inability to differentiate between noise and subtle, yet significant variation in data leads to their vulnerability to adversarial perturbations that cause wrong predictions with high confidence. The study is aimed at identifying whether the algorithms inspired by biological evolution may achieve better results in cases where brittle robustness properties are highly sensitive to the slight noise. To answer this question, we introduce the new robust gradient descent inspired by the stability and adaptability of biological systems to unknown and changing environments. The proposed optimization technique involves an open-ended adaptation process with regard to two hyperparameters inherited from the generalized Verhulst population growth equation. The hyperparameters increase robustness to adversarial noise by penalizing the degree to which hardly visible changes in gradients impact prediction. The empirical evidence on synthetic and experimental datasets confirmed the viability of the bio-inspired gradient descent and suggested promising directions for future research. The code used for computational experiments is provided in a repository at https://github.com/yukinoi/bio_gradient_descent.

## Introduction

Modern machine learning algorithms can successfully tackle tough and complicated problems by taking patterns buried inside datasets to build a model with remarkable predictive capabilities [1]. However, the machine learning models fueling innovations in a variety of applications from large-scale genomic sequencing and medicine [2–6] to automated driving [7] and robotics [8] still pose serious challenges that make it difficult to fully trust and adopt them [7, 9, 10]. A lack of intelligence in these models leads to an inability to robustly differentiate between noise and subtle, but significant variation in data. If the noise in data includes intentionally small carefully crafted perturbations, which are used to generate so-called adversarial examples, the model may become vulnerable and misclassify them with high confidence [11–17]. It expectedly sets up the psychological roadblocks [7] to the widespread adoption of machine learning models in different domains [3, 7, 11, 12, 17].

Addressing the question of vulnerability, the researchers created various attacking strategies [18–23] to fool the model with adversarial examples as well as defenses [24–29] to resist them, but that has not solved the problem completely. The common approach to creating attacks is taking gradients with regard to their inputs, since gradients provide local linear approximations of models behavior [12]. Goodfellow et al. [11] explored the connection between models vulnerability and linear nature [30, 31] which allows introducing infinitesimal changes in the gradients. According to the authors’ reasoning, the limited precision of features leads to the identical interpretation on the input gradients and adversarial gradients. With well-separated classes, the models assign the same class for both the input and adversarial gradients as long as the magnitude of perturbations is less than the magnitude of the input gradients which are predefined by the precision of features. For this reason, it seems rational to penalize the large input gradients [12, 13] which are more likely to be utilized for generating adversarial examples. The gradient regularization methods employ the idea of a double backpropagation method first introduced by Drucker and Le Cun [32]: training neural networks by minimizing not only network “energy” but also the rate of energy changes with regard to the input features. Adopting such gradient regularization on a training dataset may increase robustness to adversarial perturbations embedded in an unseen dataset as much as adversarial training [12].

What is not specifically undertaken in the studies mentioned above is whether the models inspired by biological evolution [33–42] may result in better robustness to adversarial perturbations. This is the research question raised in this study. The fundamental aspects of biological intelligence, such as self-healing, evolution, and learning make biological organisms successful to survive in unknown and changing environments [33]. The stability and adaptability of biological systems strengthen the motivation for replicating the mechanisms of natural evolution in an attempt to create the models with characteristics comparable to those of biological systems.

This paper seeks to refine the discourse on robustness to adversarial noise with the bio-inspired gradient descent based on the generalized Verhulst population growth equation [43, 44]. The hyperparameters of the Verhulst equation are used to regularize gradients or, to put it more specifically, to penalize the degree to which imperceptible changes in gradients may influence prediction results. We refer to them as *the lower and upper levels of visibility* as they limit the gradients to be near zero so that any small magnitude perturbation hidden in the gradients is “visible” and, then, has no influence on prediction results.

## Materials and methods

### Gradient descent

**Fig 1:**
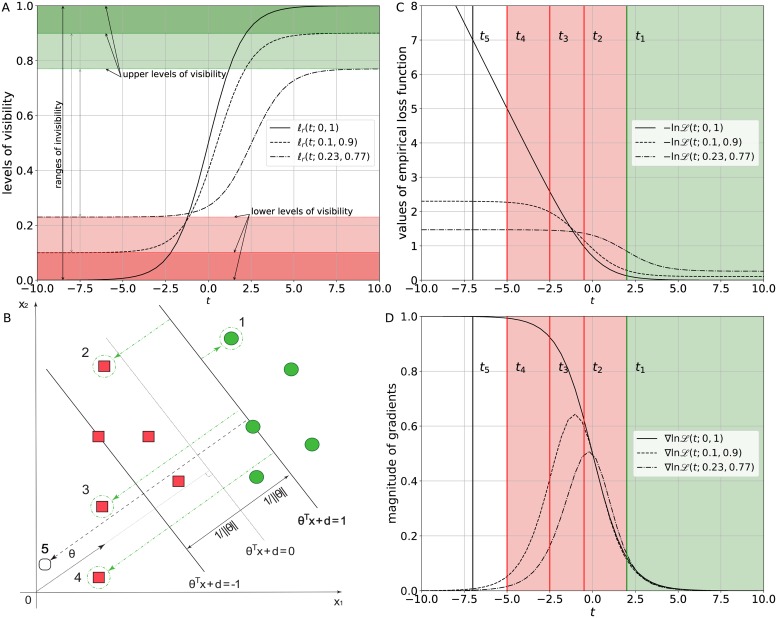
The influence of a difference in approaching the lower and the upper asymptote on the magnitude of gradients. (A) The definitions of levels of visibility and ranges of invisibility based on the generalized logistic loss function *ℓ*_*r*_(*t*; *a*, *b*) subject to the different pairs (*a*, *b*) = {(0, 1), (0.1, 0.9), (0.23, 0.77)} and *r* = 1. (B) A decision boundary with reliable (1), noisy (2,3,4), and adversarial (5) instances. (C) The empirical generalized logistic loss function −ln *ℒ*(*t*; *a*, *b*) with the levels of visibility for reliable *t*_1_, noisy *t*_2_, *t*_3_, *t*_4_, and adversarial *t*_5_ instances subject to the different pairs (*a*, *b*) = {(0, 1), (0.1, 0.9), (0.23, 0.77)} and *r* = 1. (D) The magnitude of the gradients of the empirical generalized logistic loss function ∇ln *ℒ*(*t*; *a*, *b*) subject to the different pairs (*a*, *b*) = {(0, 1), (0.1, 0.9), (0.23, 0.77)} and *r* = 1.

Similarly to the learning setting presented by Soudry et al. [45] we consider a dataset ${\left\{x_{i},y_{i}\right\}}_{i=1}^{m}$ with $x_{i}\in R^{n}$, *y*_*i*_ ∈ {0, 1} and minimize an empirical loss function

$$\begin{array}{c} L\left(\theta \right)=\sum_{i=1}^{m}\ell \left(\theta^{T}x_{i}\right), \end{array}$$

with a weight vector $\theta \in R^{n}$. We are interested in linearly separable problems with a smooth monotone strictly decreasing and non-negative loss function (see Assumption 1 and 2). For clarity, the learning setting is illustrated in Fig 1.

**Assumption 1**. *The dataset is linearly separable*: ∃ θ* *such that* ∀*i*: θ*^T^ x_*i*_ > 0.

**Assumption 2**. ∀t∈R: *ℓ*(*t*) *is a differentiable, monotonically decreasing function bounded from below*: *ℓ*(*t*)>0, *ℓ*′(*t*)<0, $\lim_{t\to \infty }\;\ell \left(t\right)=\lim_{t\to \infty }\;\ell^{\prime }\left(t\right)=0$.

The solution to the problem $\min_{\theta \in R^{n}}L\left(\theta \right)$ can be found using the *l*^*th*^ iteration of the gradient descent *GD* updates with a learning rate *η*:

$$\begin{array}{c} \theta_{l+1}=\theta_{l}-\eta \nabla L\left(\theta_{l}\right)=\theta_{l}-\eta \sum_{i=1}^{m}\ell^{\prime }\left(\theta_{l}^{T}x_{i}\right)x_{i}. \end{array}$$ (1)

In Eq (1) it is assumed that ∀*i* ∈ {1, …, *m*}: *y*_*i*_ = 1, ‖*x*_*i*_‖<1.

The function *ℓ*(*t*) describes a common classification loss function, including the exponential and logistic loss. The generalized Verhulst model allows us to introduce the generalized logistic loss function. In contrast to the simple loss function, the generalized loss function involves a lower and an upper asymptote that allow additional regulating the magnitude of input gradients.

### Generalized Verhulst growth model

The population growth *P*(*t*) in an infinite environment can be described with a linear ordinary differential equation [44, 46, 47]:

$$\begin{array}{c} \frac{dP(t)}{dt}=rP\left(t\right), \end{array}$$ (2)

where *r* ≡ *b* − *d* > 0 is the *per capita* rate of population growth, *b* and *d* are respective *per capita* rates of birth and death. The solution to this equation is

$$\begin{array}{c} P\left(t\right)=P_{0}\exp\left(rt\right), \end{array}$$ (3)

where *P*_0_ is the initial population.

Eq (2) introduces the Malthus model that describes an exponential growth of the population [47]. The Malthus model seems sufficient to describe the population in the initial stage of growth while the population is still small. However, it fails on a longer time interval since it leads to an infinite growth $\lim_{t\to \infty }\;P\left(t\right)=\infty$ as it does not consider the scarcity of resources.

To overcome this disadvantage, Verhulst assumed that Eq (2) should include the correction term [43, 46]. This term approaches 1 when *P*(*t*) tends to zero and decreases linearly as *P*(*t*) increases. Thus, the Malthus equation takes the form of the Verhulst equation that includes the correction term proportional to −*P*(*t*)^2^/*K*:

$$\begin{array}{c} \frac{dP(t)}{dt}=rP\left(t\right)(1-\frac{P(t)}{K}), \end{array}$$ (4)

where *K* is the carrying capacity that represents the maximum size of population which can be supported by the environment. As the population size approaches the carrying capacity $\lim_{t\to \infty }\;P\left(t\right)=K$, the population stops growing.

The solution to Eq (4) introduces the logistic growth model:

$$\begin{array}{c} P\left(t\right)=\frac{K}{1-(1-\frac{K}{P_{0}})\exp\left(-rt\right)}. \end{array}$$ (5)

The extension to Eq (4) that also considers a critical population size can be rewritten as

$$\begin{array}{c} \frac{dP(t)}{dt}=r(P(t)-A)(1-(\frac{P(t)-A}{K-A})); \end{array}$$ (6)

$$\begin{array}{c} P\left(t\right)=A+\frac{K-A}{1-(1-(\frac{K-A}{P_{0}+A}))\exp\left(-rt\right)}, \end{array}$$ (7)

where *K* is the upper asymptote or the population carrying capacity; *A* is the lower asymptote or the population minimum size.

The asymptote *A* indicates critical population thresholds 0 ≤ *A* < *K* below which a population crashes to extinction. It serves as a substitute for the Allee effects [48] which are broadly defined as a decline in individual fitness at low population size or density.

### Bio-inspired gradient descent

We introduce the generalized logistic loss function *ℓ*_*r*_(*t*; *a*, *b*) with regard to the generalized Verhulst growth model Eq (6) and its solution Eq (7):

$$\begin{array}{c} \ell_{r}^{\prime }\left(t;a,b\right)=r\left(\ell_{r}\left(t;a,b\right)-a\right)(1-\frac{\ell_{r}\left(t;a,b\right)-a}{b-a}) \end{array}$$ (8)

$\forall t\in R:\ell_{r}^{\prime }\left(t;a,b\right)<0,\lim_{t\to \infty }\;\ell_{r}^{\prime }\left(t;a,b\right)=\lim_{t\to -\infty }\;\ell_{r}^{\prime }\left(t;a,b\right)=0$,

$$\begin{array}{c} \ell_{r}\left(t;a,b\right)=a+\frac{b-a}{\left(1-(1-(\frac{b-a}{P_{0}+a}))\exp\left(-rt\right)\right)}, \end{array}$$ (9)

so that $\forall t\in R:\ell_{r}\left(t;a,b\right)>0,\lim_{t\to \infty }\;\ell_{r}\left(t;a,b\right)=b-a,\lim_{t\to -\infty }\;\ell_{r}\left(t;a,b\right)=a$, where the lower asymptote *a* ≡ *A*, the upper asymptote *b* ≡ *K*, 0 ≤ *a* < *b* ≤ 1 and $P_{0}+a=\frac{b-a}{2}$, from which $P_{0}=\frac{b-3a}{2}$.

Eqs (8) and (9) can be presented as

$$\begin{array}{c} \ell_{r}^{\prime }\left(t;a,b\right)=\frac{\left(b-a)r\exp(-rt\right)}{1+\exp(-rt)}=\left(b-a\right)r\ell^{\prime }\left(t\right), \end{array}$$ (10)

$$\begin{array}{c} \ell_{r}\left(t;a,b\right)=a+\frac{b-a}{1+\exp(-rt)}, \end{array}$$ (11)

where *ℓ*′(*t*) = *ℓ*(*t*)(1 − *ℓ*(*t*)) is the derivative of the simple logistic loss function *ℓ*(*t*) = *ℓ*_1_(*t*;0, 1).

Using Eq (10) to determine the gradient in Eq (1) allows us to present the bio-inspired gradient descent *BioGD* updates:

$$\begin{array}{c} \theta_{l+1}=\theta_{l}-\eta \sum_{i=1}^{m}\ell_{r}^{\prime }\left(\theta_{l}^{T}x_{i};a,b\right)x_{i}=\theta_{l}-\eta_{r}\sum_{i=1}^{m}\ell^{\prime }\left(\theta_{l}^{T}x_{i}\right)x_{i}, \end{array}$$ (12)

where *η*_*r*_ = (*b* − *a*)*rη*.

For the sake of completeness, we introduce the empirical generalized logistic loss function and its gradient as follows:

$$\begin{array}{c} \text{ln}\;L\left(\theta ;a,b\right)=-\sum_{i=1}^{m}y_{i}\ln\ell_{r}\left(\theta^{T}x_{i};a,b\right)+\left(1-y_{i}\right)\ln(1-\ell_{r}\left(\theta^{T}x_{i};a,b\right)), \end{array}$$

$$\begin{array}{c} \nabla \ln L\left(\theta ;a,b\right)=-\sum_{i=1}^{m}\left(y_{i}-\ell_{r}\left(\theta^{T}x_{i};a,b\right)\right)\frac{\ell_{r}^{\prime }\left(\theta^{T}x_{i};a,b\right)}{\left(1-\ell_{r}\left(\theta^{T}x_{i};a,b\right)\right)\ell_{r}\left(\theta^{T}x_{i};a,b\right)}x_{i},\forall y_{i}\in \left\{0,1\right\}. \end{array}$$

Then, taking into account our assumption that *y*_*i*_ = 1, Eq (12) can be given as:

$$\begin{array}{c} \theta_{l+1}=\theta_{l}-\eta \sum_{i=1}^{m}\frac{\ell_{r}^{\prime }\left(\theta_{l}^{T}x_{i};a,b\right)}{\ell_{r}\left(\theta_{l}^{T}x_{i};a,b\right)}x_{i}=\theta_{l}-\eta_{r}\sum_{i=1}^{m}\frac{\ell^{\prime }\left(\theta_{l}^{T}x_{i}\right)}{\ell_{r}\left(\theta_{l}^{T}x_{i};a,b\right)}x_{i}. \end{array}$$ (13)

The *BioGD*’s gradients in Eq (13) involve the derivative of the logistic loss function $\ell^{\prime }\left(\theta_{l}^{T}x_{i}\right)$ which is directly used to define the Hessian matrix of the simple logistic loss function in a second-order gradient method. This means that we can regularize the first-order gradients by partly employing the beneficial properties of the second-order method.

By definition, *b* − *a* ≤ 1 that implies $P_{0}\leq \frac{1}{2}-a$. Let a*(*b*) be the solution to *f*(a*(*b*);*b*) = 0, where $f\left(a;b\right)=\frac{b-3a}{2}$. Then, *P*_0_ < *a* if *a* > a*(*b*). This condition guarantees that the lower asymptote of the generalized logistic loss function *ℓ*_*r*_(*t*; *a*, *b*) is approached much more gradually than the upper asymptote. In other words, the lower plateaus of the generalized logistic loss function *ℓ*_*r*_(*t*; *a*, *b*) with the nontrivial values (*a*, *b*) = {(0.1, 0.9), (0.23, 0.77)} are broader than its upper plateaus in comparison with the simple logistic loss function *ℓ*(*t*) (see Fig 1A).

The solution a*(*b*) influences the rate of the loss function growth *ℓ*_*r*_(*t*; *a*, *b*) if *t* < 0. The value of the lower asymptote has impact on the robustness of gradient descent if ∀*i* ∈ {1, …, *m*}: *t* = θ*^T^ x_*i*_ < 0, i.e. in the presence of noisy and adversarial labels. Fig 1B depicts a decision boundary with reliable **1**, noisy **2**, **3**, **4**, and adversarial **5** instances. The circles denoted by **1** are true positive instances. The squares present true negative instances. The instances indexed by **2**, **3**, **4**, but annotated as a positive class, are noisy labels. An adversarial instance denoted by **5** is moved away from its legitimate class in the direction orthogonal to **θ** and, as a result, is “invisible” and misclassified by the model.

The labels marked in Fig 1B specify the reliable (*t* ≥ 0) and noisy (*t* ≤ 0) regions for the empirical logistic loss function and its gradient in Fig 1C and 1D. As it can be seen, the difference in approaching the lower and upper levels of visibility produces a positive effect on the loss function by dropping noisy *t*_1_, *t*_2_, *t*_3_, *t*_4_ and adversarial *t*_5_ instances. In addition, it limits the gradients to be near zero that allows any small magnitude perturbations embedded in the gradients to be “visible” and make sure that they have no influence on classification results.

Therefore, the existing difference in approaching the two asymptotes allows us to expose small magnitude perturbations below some lower and upper asymptotes, i.e. the lower and upper levels of visibility, and diminish its negative effect on results. In terms of population dynamics, the range of invisibility between these levels, i.e. the minimum and the maximum population size may be interpreted as the population persistence in spite of very limited food supply or space.

### BioGD

*BioGD* represents an implementation of bio-inspired gradient descent (see Algorithm). The algorithm involves the optimization of hyperparameters *a*, *b* and *r* with a grid search. A grid with sufficient granularity to optimizing hyper-parameters can be easily implemented but requires considerable computational costs if there is a need for smaller grid step sizes. In addition, the hyperparameters need to be optimized with constraints imposed by their bio-inspired interpretation in terms of the generalized Verhulst equation. An inappropriate choice of grid steps taken by the constrained optimization problem may lead to a lack of convergence. As an alternative to grid search, a random search may be taken into consideration [49]. This technique combines the hyperparameters randomly and demonstrates comparatively better results. However, as the method is entirely random, it leads to high variance. Bayesian optimization has lower variance as it searches the hyperparameters relying on probabilistic inference [50]. This virtue may, however, induce bias in the estimates of hyperparameters in the presence of outliers. Consequently, the choice of method depends on specific requirements and hyperparameter space complexity related to the problem to be solved. In this paper, we focus on the problem of robustness to outliers, in particular, adversarial noise. For this reason, we opt for a grid search to provide more reliable results by the exclusion of unnecessary variance in the estimates of hyperparameters.

**Algorithm** Bio-inspired gradient descent

1: **procedure** BioGD (x, *y*, *η*, *n*)

2:  Initialize **θ**_0_;

3:  Initialize *a* ∈ [*a*_min_, *a*_max_], *b* ∈ [*b*_min_, *b*_max_], *r* ∈ [*r*_min_, *r*_max_];

4:  Initialize a grid of *n* points in the space *a* × *b* × *r*;

5:  Split (x, *y*) into train (x, *y*)_*T*_ and cross-validation (x, *y*)_*CV*_ subsets;

6:  *l* ← 0;

7:  **repeat**

8:   $\theta_{l+1}\leftarrow \theta_{l}-(b-a)r\eta \nabla_{\left(\theta \right)}L(\theta_{l},{\left(\text{x},y\right)}_{T})$;

9:   *l* ← *l* + 1;

10:  **until** converge

11:  $\left(a,b,r)\leftarrow GridSearch(L(\theta_{l+1},{\left(\text{x},y\right)}_{CV}),a,b,r\right)$;

12:  **return** **θ**_*l*+1_, (*a*, *b*, *r*)

## Computational experiments

We analyzed the impact of the hyperparameters on the robustness properties empirically in order to support the results of theoretical outcomes. According to theoretical results, the hyperparameters *a* and *b*: 1) directly deal with the magnitude of gradients; 2) are adaptive to the newly updated data; 3) allow us to improve robustness to noise by penalizing the degree to which hardly visible changes in gradients impact prediction. Therefore, the empirical evidence is based on the comparison between the simple logistic loss with a = 0, b = 1 and the generalized logistic loss with a_opt_, b_opt_. In addition, we varied incrementally a number of instances *m* to explore the adaptivity of the hyperparameters a_opt_, b_opt_ to the newly updated data. Finally, we justified the improvement in robustness and performance of the generalized model over the simple model on the synthetic linearly separable dataset with different proportions of noisy labels. Then, we assessed the viability of the generalized logistic loss with the hyperparameters in the more realistic setting on experimental datasets. The chosen datasets are normally not linearly separable but are indicative of the behavior of the hyperparameters and their influence on prediction results.

BioGD suggests a predefined value for the rate of population growth for computational experiments on synthetic and experimental datasets. There are two reasons for choosing this value. First, we intend to analyze the direct influence of the hyperparameters *a* and *b* on the magnitude of gradients. Second, we explore the benefits of the generalized logistic loss with some optimal hyperparameters a_opt_ and b_opt_ over the simple logistic loss with a = 0 and b = 1. The simple logistic loss lacks the parameter *r* by definition. As a consequence, it is reasonable to choose *r* = 1.

While the hyperparameters *a* and *b* address the issue of robustness, the growth rate *r* is aimed at accelerating the rate of convergence adaptively. Consequently, the hyperparameter *r* does not play a crucial role in improving the results on the synthetic and experimental datasets since the results of computational experiments are given from the perspective of a discriminative model such as logistic regression for binary classification. This normally implies processing smaller datasets. Even if the detailed analysis of the convergence rate issue is beyond the scope of this study, we still took the parameter *r* into consideration to show the usefulness and applicability while applying the proposed technique to a more complicated model on a large dataset for multiclass image classification.

### Synthetic datasets

#### Design of experiments

We created an *n*-dimensional dataset so that each dimension’s mean *μ*_*j*_ is sampled from a Gaussian distribution N(0,1) while each dimension’s standard deviation *σ*_*j*_ is generated according to a distribution N(1,1). The feature space of n-dimensional instances is sampled from the distribution $N(\mu_{j},\sigma_{j})$. The instances in the dataset were labelled by a randomly chosen hyperplane, so that the generated dataset is linearly separable. A similar design of experiments is given by [51]. For the originally generated dataset, the portion of mislabelled examples is *p* = 0.0. We constructed the noisy versions of this dataset by flipping the labels for the randomly selected proportion of data instances *p* = {0.05, 0.1, 0.15, 0.2, 0.25, 0.3}. A more detailed description of this procedure is presented by [52] The experiments were carried out by changing the number of instances incrementally *m*_*k*_ ∈ [20, 100] and setting the number of features *n* = 10.

The datasets were divided into the training subset and the validation subset using 5-fold cross-validation. We evaluated the cross-validation estimates of the hyperparameters a_opt_ and b_opt_ with BioGD refined over a grid of 20 points in the hyperparameter space *a* × *b*, where *a* ∈ [0, 0.15] and *b* ∈ [0.85, 1]. In addition, we computed the mean and standard deviation (sd) of empirical logistic loss $\text{ln}\;L(\theta ;x^{m_{k}},a_{\text{opt}},b_{\text{opt}})$ over *N* = 200 experiments to guarantee the reliable estimates. We used the default value for the learning rate *η* = 1.5e-8 to run BioGD.

#### Results

**Fig 2:**
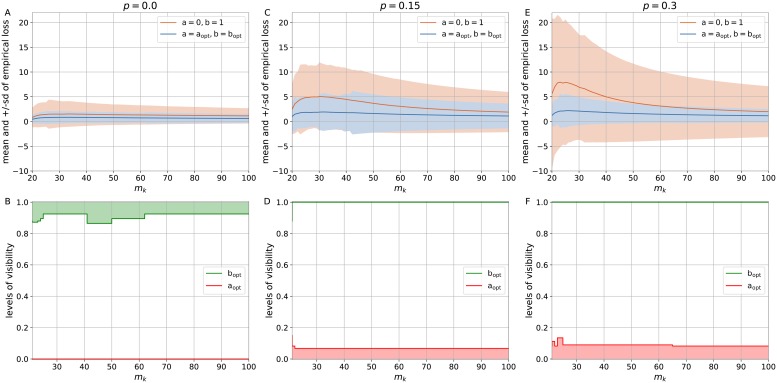
The estimates for the simple logistic loss with a = 0, b = 1 and the generalized logistic loss with the optimal levels of visibility a_opt_, b_opt_ subject to *m*_*k*_ ∈ [20, 100] and *p* = {0.0, 0.15, 0.3}. (A) The mean and +/-sd of empirical loss subject to *p* = 0.0. (B) The levels of visibility a_opt_, b_opt_ subject to *p* = 0.0. (C) The mean and +/-sd of empirical loss subject to *p* = 0.15. (D) The levels of visibility a_opt_, b_opt_ subject to *p* = 0.15. (E) The mean and +/-sd of empirical loss subject to *p* = 0.3. (F) The levels of visibility a_opt_, b_opt_ subject to *p* = 0.3.

Fig 2A, 2C and 2E present the estimates of mean and sd of empirical loss for the simple logistic loss with a = 0, b = 1 and the generalized logistic loss with the optimal levels of visibility a_opt_, b_opt_ with regard to *m*_*k*_ = [20, 100] and *p* = {0.0, 0.15, 0.3}. As it can be seen, the generalized logistic loss with the asymptotes a_opt_, b_opt_ brings the benefits to both mean and sd of the empirical loss. Moreover, increasing the portion of noisy labels up to *p* = 0.3 (see Fig 2E) allows us to have distinct advantage of introducing the loss with optimal levels of visibility a_opt_, b_opt_ over the simple loss with a = 0, b = 1 on linearly separable dataset *p* = 0.0 (see Fig 2A). The impact of levels of visibility on the estimates may be seen in Fig 2B, 2D and 2F. We have linearly separable datasets in Fig 2B. The lower level of visibility does not come into play here as a_opt_ = 0, but the upper level of visibility handles premature convergence of the empirical loss. On the other hand, different portions of noisy labels were presented in Fig 2D and 2F, where we can observe how the lower level of visibility minimizes both the mean and sd of the empirical loss. If the lower level of visibility a_opt_ results in larger values, it produces a more dramatic effect on the estimates of empirical loss. This is expected by definition as the lower level allows us to directly regulate the magnitude of gradients (see Fig 1D). The results of experiments for different pairs *p* = {0.05, 0.1} and *p* = {0.2, 0.25} are given in S1 and S2 Figs, respectively.

**Fig 3:**
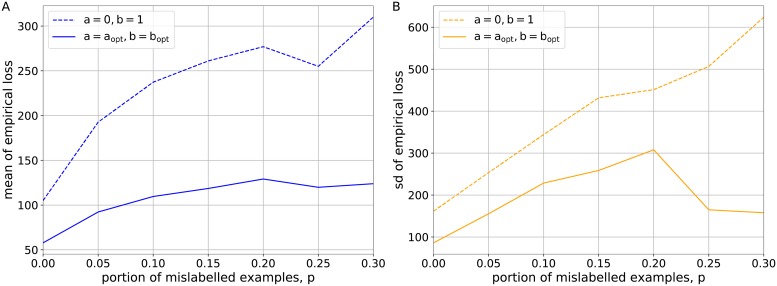
The estimates for the simple logistic loss with a = 0, b = 1 and the generalized logistic loss with the optimal levels of visibility a_opt_, b_opt_ subject to the proportions of noisy labels *p* = {0.0, 0.05, 0.1, 0.15, 0.2, 0.25, 0.3}. (A) The mean of empirical loss summed over the interval *m*_*k*_ = [20, 100]. (B) The sd of empirical loss summed over the interval *m*_*k*_ = [20, 100].

To confirm the viability of introducing the levels of visibility, we computed the estimates summed over the interval *m*_*k*_ = [20, 100] for the simple logistic loss with a = 0, b = 1 and the generalized logistic loss with the optimal levels of visibility a_opt_, b_opt_ with regard to different proportions *p* = {0.0, 0.05, 0.1, 0.15, 0.2, 0.25, 0.3} (see Fig 3). As we can see, there is a more noticable improvement in both the mean (see Fig 3A) and sd (see Fig 3B) of the empirical loss for larger portion of noisy labels that fully comply with the theoretical reasoning behind the definitions of visibility levels presented in Fig 1. The estimates of mean and +/-sd of empirical loss for *p* = {0.0, 0.3} are given in S1 and S2 Files, respectively.

We used the Wilcoxon signed-rank test [53] to confirm the statistical significance of the differences in mean against sd of the empirical loss between the generalized logistic loss and the simple logistic loss without assuming them to follow the normal distribution. This test is usually considered an alternative to the paired Student’s t-test if the population can not be assumed to be normally distributed. The null hypothesis is that there is no improvement in the mean and sd of the empirical loss for the generalized logistic loss with the optimal levels of visibility over the empirical loss for the simple logistic loss. Since the p-value turns out to be 0.01563, i.e. less than the 0.05 significance level, we rejected the null hypothesis. Consequently, the difference between the mean and sd of the empirical loss for the generalized logistic loss and the simple loss is statistically significant. In addition, we applied a one-sided Wilcoxon test with Bonferroni correction [54] for each *p*. The tests resulted in p-value<0.001 which means that for each value of *p* we have a sufficient reason to reject the null hypothesis.

### Experimental datasets

#### Design of experiments

We chose 11 experimental datasets, which are freely available from UCI Machine Learning repository, for validating BioGD. The information given in Table 1 includes the number of instances *m*, the number of features *n*, the class distribution |*y*_*i*_ = 0| and |*y*_*i*_ = 1|. Incrementally varying a number of instances *m*_*k*_ ∈ [20, 100] randomly taken from the original dataset with number of instances *m*, we explored the adaptivity of the hyperparameters a_opt_ and b_opt_ to the newly updated data. By analogy with the empirical setting for the synthetic datasets, we implemented BioGD over a grid of 20 points in the hyperparameter space *a* × *b*, where *a* ∈ [0, 0.15] and *b* ∈ [0.85, 1], to make the cross-validation estimates of the empirical loss and the asymptotes. The default learning rate *η* = 1.5e-8 was used.

**Table 1:** A brief description of the experimental datasets.

Dataset	*m* = |*y*_*i*_|	*m* = |*y*_*i*_ = 0|	*m* = |*y*_*i*_ = 1|	*n*
Blood Transfusion Service Center (blood)	748	570	178	4
Banknote Authentication (banknote)	1372	762	610	4
Vertebral Column (vertebral)	309	100	209	6
Liver Disorder (liver)	345	145	200	6
Pima Indians Diabetes (pima)	768	500	268	8
Breast Cancer Wisconsin (breast-win)	683	444	239	9
Heart Statlog (heart)	270	150	120	13
Climate Model Simulation Crashes (climate)	540	46	494	17
Parkinsons (parkinsons)	195	48	147	22
Chronic Kidney Disease (chronic-kidney)	157	114	43	24
Cervical Cancer (cervical)	668	623	45	33

#### Results

**Fig 4:**
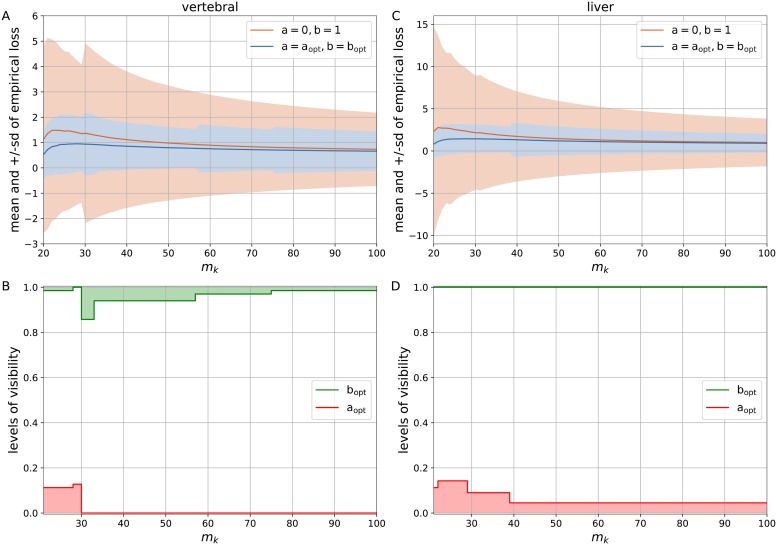
The estimates for the simple logistic loss with a = 0, b = 1 and the generalized logistic loss with the optimal levels of visibility a_opt_, b_opt_ subject to *m*_*k*_ ∈ [20, 100]. (A) The mean and +/-sd of empirical loss for the *vertebral* dataset. (B) The levels of visibility a_opt_, b_opt_ for the *vertebral* dataset. (C) The mean and +/-sd of empirical loss for the *liver* dataset. (D) The levels of visibility a_opt_, b_opt_ for the *liver* dataset.

**Fig 5:**
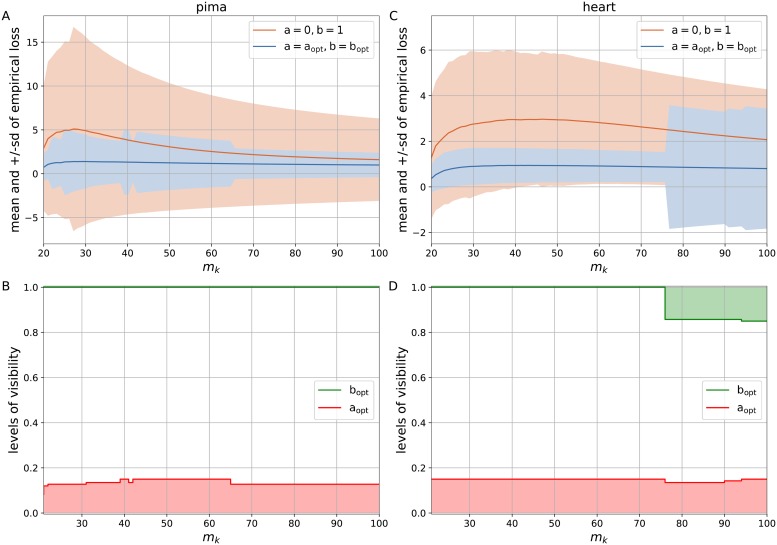
The estimates for the simple logistic loss with a = 0, b = 1 and the generalized logistic loss with the optimal levels of visibility a_opt_, b_opt_ subject to *m*_*k*_ ∈ [20, 100]. (A) The mean and +/-sd of empirical loss for the *pima* dataset. (B) The levels of visibility a_opt_, b_opt_ for the *pima* dataset. (C) The mean and +/-sd of empirical loss for the *heart* dataset. (D) The levels of visibility a_opt_, b_opt_ for the *heart* dataset.

According to the results of computational experiments, the used datasets can be divided into the groups with marked, little and no improvement in the generalized logistic loss with the optimal levels of visibility a_opt_, b_opt_ over the simple logistic loss with a = 0, b = 1. Figs 4 and 5 demonstrate the estimates of mean and sd of the empirical loss over *N* = 200 experiments with regard to *m*_*k*_ = [20, 100] for *vertebral*, *liver*, *pima*, and *heart*. We can observe that the generalized logistic loss with the asymptotes a_opt_, b_opt_ brings the benefits to both mean and sd of the empirical loss. Moreover, the loss function converges with an increasing number of examples *m*_*k*_. As we have seen in case of the synthetic dataset, the variations of the upper level of visibility b_opt_ can be attributed to premature convergence of the empirical loss function, while the lower level of visibility is sensitive to the outliers that results in its divergence. Moreover, the larger values for either the lower or upper level of visibility are indicative of the domination of the above-mentioned issues.

However, for *banknote*, *breast-win*, and *climate* datasets, we obtained little improvement as both levels of visibility influence the results or their impact is negligible (see S3 Fig). This pinpoints a lack of salient presence of one of the above-mentioned issues. As a result, the empirical loss function may not converge with an increasing number of examples *m*_*k*_. The levels of visibility may not produce any improvement (see S4 Fig). For *blood* dataset the curves of empirical loss function for the generalized logistic loss with the optimal levels of visibility a_opt_, b_opt_ and the simple logistic loss with a = 0, b = 1 have been overlapped. For *parkinsons* dataset, the empirical loss functions have little advantage over the generalized loss. Both almost overlap. In S5 Fig the empirical loss functions does not converge for the simple logistic loss with a = 0, b = 1 at all. The corresponding curves have been set as zero-valued. But the optimal levels of visibility a_opt_, b_opt_ still allow us to estimate the mean and sd of the generalized loss.

**Fig 6:**
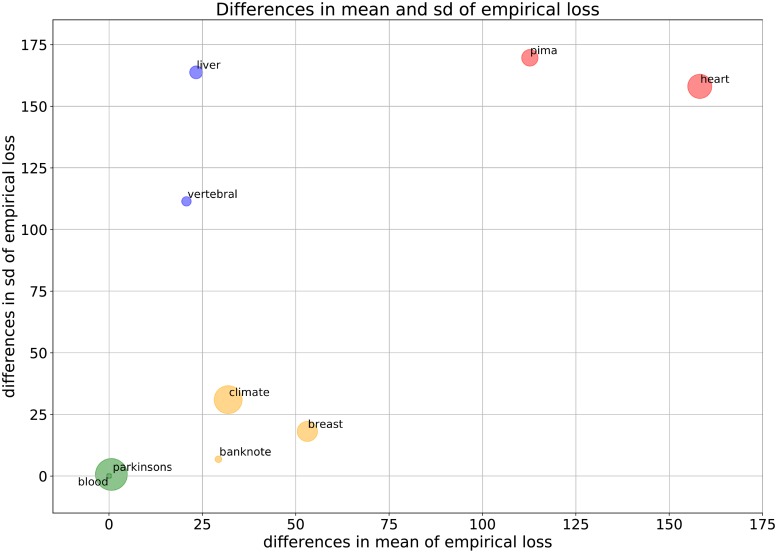
The differences in mean and sd of empirical loss between the simple logistic loss with a = 0, b = 1 and the generalized logistic loss with the optimal levels of visibility a_opt_, b_opt_ for all the datasets summed over the interval *m*_*k*_ ∈ [20, 100].

Fig 6 depicts the differences in mean against sd of the empirical loss between the simple logistic loss and the generalized logistic loss for all the datasets summed over the interval *m*_*k*_ ∈ [20, 100]. The circles, radius of which is proportional to the number of features *n* (see Table 1), are colored according to the rate of improvement on the mean and sd of empirical loss. As the empirical loss function does not converge for *chronic-kidney* and *cervical* datasets in case of a = 0, b = 1, the illustrated differences do not include these datasets. The estimates of mean and sd for the simple loss and the generalized loss separately are given in S6 Fig.

By analogy with the synthetic datasets, we used the Wilcoxon signed-rank test to confirm the statistical significance of the differences in mean against sd of the empirical loss between the simple logistic loss and the generalized logistic loss. Since the p-value is equal to 0.01427, i.e. less than the 0.05 significance level, we rejected the null hypothesis. The difference between the mean and sd of the empirical loss for the generalized logistic loss and the simple loss is statistically significant.

### Real-world application

#### Design of experiments

As an example of the applicability of the proposed optimization technique, we applied it to an Adam-based [55] neural network with two hidden layers, each of them containing 10 neurons. Each layer was activated by an advanced sigmoid function. The activation function of the neural network was built with the generalized logistic loss that introduced the hyperparameters a, b, and r into the model. We used the default values for the Adam optimizer: *η* = 0.001, *β*_1_ = 0.9, *β*_2_ = 0.999. The neural network was trained on the freely available MNIST handwritten digits dataset [56] to solve a 10-class classification problem. The dataset consists of 10 handwritten digits {0, .., 9} from 250 different people, 50% of high school students, and 50% of employees from the Census Bureau. Each feature vector contains *n* = 784 pixels unrolled from the original 28x28 pixels images. The dataset consisted of *m* = 60000 examples for training and *m* = 10000 examples for testing. The training set was divided into an actual training set of 50000 examples and 10000 validation examples for selecting the hyperparameters. The number of epochs and the batch size for training were n_epoch_ = 1 and n_batch_ = 25, respectively. The hyperparameters were optimized with a random search as a less computationally expensive alternative to a grid search in the hyperparameter space *a* × *b* × *r*, where *a* ∈ [0, 0.15], *b* ∈ [0.85, 1], and *r* ∈ [0.5, 5.1]. We tested the model on a subset with adversarial perturbations to confirm the importance of the hyperparameters for regulating the degree to which changes in gradients impact prediction.

The model of neural network was built in TensorFlow using Keras API. We also implemented a random search with a random pick 15% of the permutations for optimizing the hyperparameters with Talos and FGSM [11] for generating gradient-based attacks with Foolbox [57]. FGSM allowed us to add the signs of gradients to the images, and, by that, increase the magnitude until the images were misclassified.

#### Results

**Fig 7:**
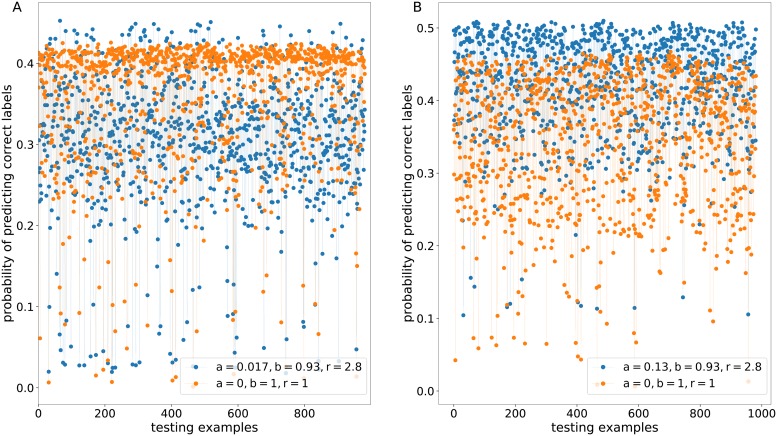
The probabilities of predicting correct labels in the presence of adversarial perturbations for the advanced sigmoid activation function with the hyperparameters a_opt_, b_opt_, r_opt_ and the simple sigmoid activation function with the hyperparameters a = 0, b = 1, r = 1. (A) The hyperparameters are equal to a_opt_ = 0.017, b_opt_ = 0.93, r_opt_ = 2.8. (B) The hyperparameters are equal to a_threshold_ = 0.13, b_opt_ = 0.93, r_opt_ = 2.8.

Fig 7 depicts the probabilities of predicting correct images on a testing dataset in presence of adversarial noise for the advanced sigmoid activation function and the simple activation function. We can see that the advanced function with the optimal hyperparameters did not bring distinct advantages (see Fig 7A) but still allowed us to guarantee higher accuracy that is 95.6% in comparison with the simple function that gives 92.8% on a testing dataset without adversarial examples.

By definition, the hyperparameter a allows us to regulate the magnitude of gradients. Consequently, we set a threshold value a_threshold_ = 0.13 that was used on training the neural network model. The neural network model with the parameter a_threshold_ instead of a_opt_ still showed better prediction on a testing dataset that does not contain adversarial noise—94.6% against 92.8%. We investigated the influence of this parameter on the probability of predicting correct labels. The values b and r were fixed for clarity. Fig 7B) reflects a more marked increase in the probabilities of predicting correct labels for the advanced activation function in comparison with the simple activation function.

Consequently, the proposed optimization technique may contribute to increasing robustness to adversarial noise by penalizing the degree to which subtle changes in gradients impact prediction.

## Discussion

BioGD shares some similarity with the gradient regularization method presented by Ross and Doshi-Velez [12] which allows regulating how much infinitesimal noise in gradients affect predictions. However, Ross and Doshi-Velez proposed a second-order method which implies a cost of taking second derivatives. Yu et al. [13], in turn, proposed a novel robust training method by regulating the first-order gradients of neural networks. But, the adversarial examples and neural networks were considered as quasi-linear models.

The BioGD is the first-order gradient method which aims to solve a predefined optimization problem, but also involves an open-ended adaptation process with regard to two hyperparameters. Considering the fact that these hyperparameters directly deal with vulnerable gradients, are adaptive to the newly updated data, and allow us to improve robustness to adversarial noise by regularizing the degree to which hardly visible changes in gradients impact prediction, the BioGD algorithm seems to be an efficient solution to the posed problem.

There are two important limitations to this research. First, the BioGD is based on the Verhulst logistic growth equation. An alternative model of logistic growth is the Gompertz equation that shows more flexible behavior near the lower and upper asymptotes in comparison with the Verhulst equation. Consequently, it seems promising to explore the effect of the asymptotes of the Gompertz equation on the magnitude of gradients below the lower and upper levels of visibility.

The other limitation is related to the open-ended adaptation process of the pair of hyperparameters. The implemented hyperparameter optimization controls the behavior of a learning algorithm using the empirical loss on a validation subset. Optimizing the hyperparameters on the same dataset tunes only the values of this pair. This adaptation process may be interpreted in terms of the single species population growth. As a direction for future research, we suggest adapting BioGD to a meta-learning framework that infers a learning algorithm from a set of datasets in order to improve robustness on unseen datasets. In contrast to hyperparameter optimization, meta-learning would imply optimizing a set of the pairs of hyperparameters and may be considered in the context of multiple species population growth.

## Supporting information

S1 File The estimates of mean and +/-sd of empirical loss subject to *m*_*k*_ ∈ [20, 100] and *p* = 0.0. (CSV)

S2 File The estimates of mean and +/-sd of empirical loss subject to *m*_*k*_ ∈ [20, 100] and *p* = 0.3. (CSV)

S1 Fig The estimates for the simple logistic loss with a = 0, b = 1 and the generalized logistic loss with the optimal levels of visibility a_opt_, b_opt_ subject to *m*_*k*_ ∈ [20, 100] and *p* = {0.05, 0.1}. (A) The mean and +/-sd of empirical loss subject to *p* = 0.05. (B) The levels of visibility a_opt_, b_opt_ subject to *p* = 0.05. (C) The mean and +/-sd of empirical loss subject to *p* = 0.1. (D) The levels of visibility a_opt_, b_opt_ subject to *p* = 0.1.
(EPS)

S2 Fig The estimates for the simple logistic loss with a = 0, b = 1 and the generalized logistic loss with the optimal levels of visibility a_opt_, b_opt_ subject to *m*_*k*_ ∈ [20, 100] and *p* = {0.2, 0.25}. (A) The mean and +/-sd of empirical loss subject to *p* = 0.2. (B) The levels of visibility a_opt_, b_opt_ subject to *p* = 0.2. (C) The mean and +/-sd of empirical loss subject to *p* = 0.25. (D) The levels of visibility a_opt_, b_opt_ subject to *p* = 0.25.
(EPS)

S3 Fig The estimates for the simple logistic loss with a = 0, b = 1 and the generalized logistic loss with the optimal levels of visibility a_opt_, b_opt_ subject to *m*_*k*_ ∈ [20, 100]. (A) The mean and +/-sd of empirical loss for the *banknote* dataset. (B) The levels of visibility a_opt_, b_opt_ for the *banknote* dataset. (C) The mean and +/-sd of empirical loss for the *breast-win* dataset. (D) The levels of visibility a_opt_, b_opt_ for the *breast-win* dataset. (E) The mean and +/-sd of empirical loss for the *climate* dataset. (F) The levels of visibility a_opt_, b_opt_ for the *climate* dataset.
(EPS)

S4 Fig The estimates for the simple logistic loss with a = 0, b = 1 and the generalized logistic loss with the optimal levels of visibility a_opt_, b_opt_ subject to *m*_*k*_ ∈ [20, 100]. (A) The mean and +/-sd of empirical loss for the *blood* dataset. (B) The levels of visibility a_opt_, b_opt_ for the *blood* dataset. (C) The mean and +/-sd of empirical loss for the *parkinsons* dataset. (D) The levels of visibility a_opt_, b_opt_ for the *parkinsons* dataset.
(EPS)

S5 Fig The estimates for the simple logistic loss with a = 0, b = 1 and the generalized logistic loss with the optimal levels of visibility a_opt_, b_opt_ subject to *m*_*k*_ ∈ [20, 100]. (A) The mean and +/-sd of empirical loss for the *chronic-kidney* dataset. (B) The levels of visibility a_opt_, b_opt_ for the *chronic-kidney* dataset. (C) The mean and +/-sd of empirical loss for the *cervical* dataset. (D) The levels of visibility a_opt_, b_opt_ for the *cervical* dataset.
(EPS)

S6 Fig The estimates for all the datasets summed over the interval *m*_*k*_ ∈ [20, 100]. (A) The mean of empirical loss against the sd of empirical loss for the simple logistic loss with a = 0, b = 1. (B) The mean of empirical loss against the sd of empirical loss for the generalized logistic loss with the optimal levels of visibility a_opt_, b_opt_.
(EPS)
